# Silent Threats of the Heart: A Case Series and Narrative Review on Suicide Left Ventricle Post-Aortic Valve Replacement in Patients with Dynamic LVOT Obstruction and Aortic Stenosis

**DOI:** 10.3390/jcm13185555

**Published:** 2024-09-19

**Authors:** Silvia Romano, Emilio D’Andrea, Dan Alexandru Cozac, Maria Teresa Savo, Antonella Cecchetto, Anna Baritussio, Marika Martini, Massimo Napodano, Barbara Bauce, Valeria Pergola

**Affiliations:** 1 Department of Cardiac, Thoracic and Vascular Sciences and Public Health, University of Padova, 35128 Padova, Italymariateresa.savo@studenti.unipd.it (M.T.S.); antonella.cecchetto@aopd.veneto.it (A.C.); anna.baritussio@aopd.veneto.it (A.B.); valeria.pergola@gmail.com (V.P.); 2 Doctoral School of the University of Medicine, Pharmacy, Science and Technology of Targu Mures, 540136 Targu Mures, Romania; 3 Cardiology Unit, Cardio-Thoracic-Vascular, and Public Health Department, Padova University Hospital, 35128 Padova, Italy

**Keywords:** aortic stenosis, hypertrophic left ventricle, multidisciplinary management, suicide left ventricle

## Abstract

Aortic stenosis (AS) is the most prevalent valvular heart disease in Europe and North America, with transcatheter aortic valve implantation (TAVI) revolutionizing its management. Hypertrophic left ventricle (HLV) frequently coexists with AS, complicating treatment due to the associated risk of left ventricular outflow tract (LVOT) obstruction, heart failure, and sudden death. A rare but severe post-aortic valve replacement (AVR) complication, termed “suicide left ventricle” (SLV), has emerged, necessitating further study. This report synthesizes current literature on SLV, its pathophysiology, and management strategies, alongside four patient case studies. The patients aged 79–87 years, underwent AVR for symptomatic AS with HLV. Post-AVR, all experienced severe complications, including dynamicLVOT gradients, systolic anterior motion (SAM) of the mitral valve, and severe hypotension, leading to death in two cases. One patient survived following surgical aortic valve replacement (SAVR) with surgical myectomy. One patient survived after TAVI. These cases highlight the critical importance of multidisciplinary Heart Team evaluations and personalized treatment plans in managing SLV. Despite advancements in AVR, SLV remains a complex, life-threatening condition, requiring an exhaustive and multifaceted approach for optimal patient outcomes. This report offers valuable insights into SLV occurrence and management from a clinical perspective.

## 1. Introduction

Aortic stenosis (AS) is the most prevalent valvular heart disease in Europe and North America, with its occurrence anticipated to rise as the population ages [1]. Transcatheter aortic valve implantation (TAVI) has revolutionized the management of AS, being one of the leading transcatheter structural interventions worldwide, especially in patients who are not candidates for surgical aortic valve replacement (SAVR) for high surgical risk [2,3].

In response to AS, the left ventricle (LV) becomes hypertrophic. Hypertrophic LV (HLV) is a complex and often severe cardiac condition characterized by the thickening of the heart explained by abnormal loading conditions, which can impede normal blood flow and lead to various complications, including heart failure, sudden cardiac death, as a result, of increased myocardial mass and associated structural and functional changes in the heart [4]. Moreover, HLV can generate dynamic left ventricular tract (LVOT) obstruction, leading to acute decompensation in response to decreased afterload, as occurs after aortic valve replacement (AVR). Therefore, the association between these two conditions should not be surprising, as HLV and AS often coexist, presenting unique challenges for treatment. Recent advancements in aortic prosthesis include several key innovations to improve patient outcomes, device durability, and procedural safety [5]. Furthermore, the procedural success of AVR in patients with HLV and AS has been promising, with significant improvements in valve function and patient symptoms [6]. However, the advent of TAVI allowed for a higher number of AVR procedures, with high complexity, but at the same time has also brought to light new complications, and a rare but serious complication known as “suicide left ventricle” (SLV) has also been observed.

This case series report and narrative review aims to synthesize the current literature on the phenomenon of SLV, its pathophysiology, clinical implications, and management strategies. We aim to report our experience with SLV following the AVR procedure. As TAVI is an evolving technique, the complications associated with it are also novel and challenging, thus we are learning from our experiences to improve the management of this specific patient population.

### 1.1. Case 1 (Figure 1)

An 87-year-old woman with known severe AS was admitted for worsening dyspnea and fatigue upon mild exertion. Her medical history included hypertension, dyslipidemia, and a previous carotid endarterectomy meanwhile, her home medical therapy included antiplatelet agents, beta-blockers, statins, and diuretics. After a Heart Team discussion, she was proposed for TAVI. The pre-procedural coronary angiography showed patent coronary arteries. The Society of Thoracic Surgeons Score (STS score) Operative Mortality was 5.58%, Morbidity & Mortality was 13.3%, and EUROscore II was 7.02%. The pre-procedural transthoracic echocardiogram (TTE) revealed a marked HLV, with an interventricular septum (IVS) of 20 mm and normal ejection fraction (EF = 58%) with no regional wall motion abnormalities. The TTE also showed a paradoxical low-flow, low-gradient type [maximum gradient 53 mmHg, mean gradient 28 mmHg, Vmax 3.6 m/s, stroke volume index (SVi) 18 mL/m^2^], with possible underestimation of gradients due to small left ventricle size. LVOT Velocity Time Integral (VTI) was not assessable due to dynamic LVOT obstruction (LVOTO) caused by systolic anterior motion (SAM) of the subvalvular mitral apparatus leading to a maximum gradient at rest up to 24 mmHg, and with a Valsalva maneuver up to 54 mmHg. There was also an associated mild mitral valve regurgitation (MVR).

**Figure 1:**
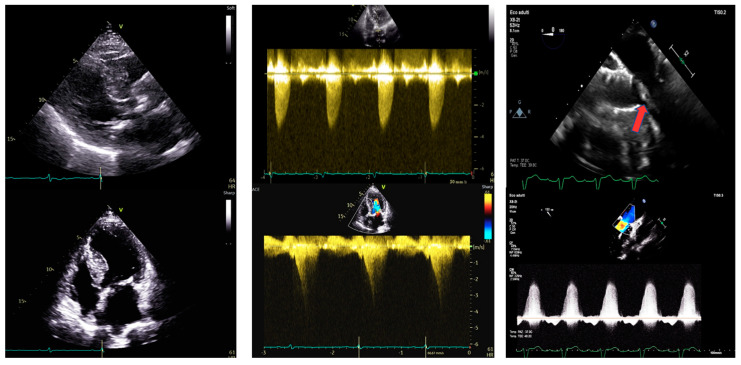
Transthoracic and transesophageal echocardiography in an 87-year-old woman with paradoxical low-flow low-gradient aortic stenosis. Left panel. PLAX view (upper) and 4-chamber view (lower). IVS telediastolic diameter = 20 mm, LVEDVi = 31 mL/m^2^. Central panel. Aortic valve peak velocity of 3.6 m/s (upper) with a mean gradient of 28 mmHg and a dynamic LVOT gradient (lower) of 54 mmHg after the Valsalva maneuver. Right panel after TAVI. SAM of the anterior mitral leaflet (upper, red arrow) by TEE causing severe mitral regurgitation. IVS: interventricular septum. LVEDVi: left ventricular end-diastolic volume indexed. LVOT: left ventricular outflow tract. SAM: systolic anterior motion.

The patient underwent TAVI with a 26 mm Evolut Pro+ bioprosthesis implantation. Upon valve deployment, the patient experienced marked hypotension (BP 60/40 mmHg). Mechanical complications such as pericardial effusion and vascular complications were ruled out. Due to severe desaturation, the patient was intubated and a temporary pacemaker was placed due to the onset of a complete atrioventricular block. Then the patient developed ventricular fibrillation, which was defibrillated with one effective DC shock. Post-procedural ventriculography showed normal left ventricular systolic function, a trans-prosthetic gradient of 20 mmHg, and the emergence of severe MVR. Post-procedural coronary angiography showed no coronary embolism in coronary arteries and no coronary ostia obstruction by the prosthesis. Aminergic support with norepinephrine, epinephrine, and dobutamine was required. The subsequent transesophageal echocardiography (TEE) confirmed the proper position of the aortic valve prosthesis, with mild paravalvular regurgitation. Significant acceleration in the LVOT (Vmax 4 m/s) was noted due to SAM of the anterior mitral leaflet (AML), with severe MVR. In the following days, aminergic support was gradually reduced. However, despite numerous attempts and no neurological complications, extubation was not possible due to concomitant acute pulmonary edema and sepsis. Percutaneous correction of the mitral defect was considered but not performed due to concurrent sepsis, patient fragility, and high risk of futility. The patient died a few days later. 

### 1.2. Case 2 (Figure 2)

An 86-year-old woman with a previous history of obstructive hypertrophic cardiomyopathy (OHCM) was admitted for worsening dyspnea (NYHA class III). Regarding her past medical history, the patient had hypertension, dyslipidemia, diabetes mellitus, and chronic obstructive pulmonary disease (COPD), and she was treated with beta-blockers, angiotensin receptor blockers (ARB), diuretics, an oral antidiabetic agent, statins, and COPD therapy. The patient presented no family history for OHCM and she refused the genetic testing. A cardiac magnetic resonance imaging (MRI) was performed, which ruled out amyloidosis and confirmed the diagnosis of OHCM. 

**Figure 2:**
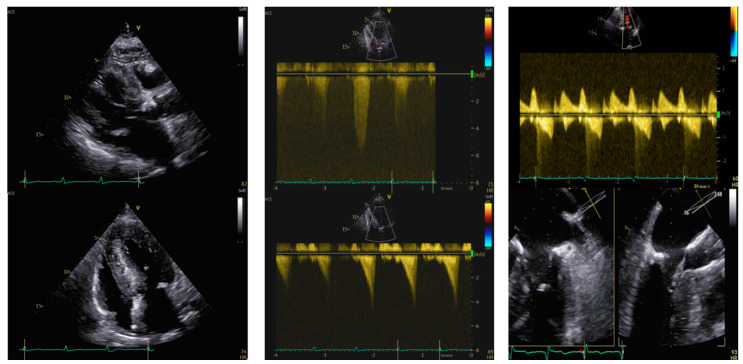
Transthoracic and transesophageal echocardiography in an 86-year-old woman before and after aortic valve stenosis percutaneous treatment and transcatheter alcohol septal ablation. Left panel: PLAX view (upper) and 4-chamber view (lower); IVS = 26 mm; LVEDVi = 45 mL/mq. Central panel: aortic valve peak velocity of 5.8 m/s (upper) with a mean gradient of 77 mmHg and dynamic LVOT gradient (lower) of 65 mmHg. Right panel—after TAVI and alcohol septal ablation: dynamic LVOT gradient (upper) of 20 mmHg but evidence of residual SAM of the anterior mitral leaflet (lower) by TEE. IVS: interventricular septum. LVEDVi: left ventricular end-diastolic volume indexed. LVOT: left ventricular outflow tract. SAM: systolic anterior motion. TAVI: transcatheter aortic valve implantation.

The pre-procedural TTE revealed a marked HLV (IVS 26 mm, posterior wall (PW) 11 mm), non-dilated, with preserved systolic function (EF 56%) and no regional wall motion abnormalities. An LVOT dynamic obstructive gradient up to 65 mmHg was found. AS was noted (maximum gradient 135 mmHg, mean gradient 77 mmHg, Vmax 5.8 m/s, continuity equation not applicable due to subvalvular gradient, planimetric area 0.7 cm^2^), along with mild MVR. Cardiac catheterization showed no coronary arteries stenosis, but a combined pulmonary hypertension. The case was collegially discussed by the Heart Team and the surgical approach was excluded due to an STS score of 9% and a EUROscore II of 8%. 

The patient underwent TAVI with a 25 mm Navitor bioprosthesis implantation. During the procedure, volume expansion and a continuous esmolol infusion of up to 50 mcg/kg/min were administered. Post-procedural left catheterization confirmed the persistence of LVOTO with a dynamic gradient of 40 mmHg. Severe MVR emerged, with increased pressures in the left ventricle. The postprocedural TTE demonstrated a gradient of 67 mmHg and a significant MVR caused by SAM of the AML. Due to the patient’s hemodynamical instability, transcatheter alcohol septal ablation was performed.

Under general anesthesia and intubation, with TEE guidance, septal branches S2 and S3 were alcoholized, and S1 was embolized using coils. Myocardial perfusion was monitored using a SonoVue echo-contrast agent. After the procedure, the LVOT gradient was reduced to 15 mmHg and MVR was trivial. The subsequent TTE confirmed a persistent subvalvular gradient of 20 mmHg and a residual SAM of the chordal structures, resulting in mild MVR. Despite the improvement in hemodynamics, the patient died a few days later due to infectious complications.

### 1.3. Clinical Case 3 (Figure 3)

A 79-year-old woman was admitted for exertional dyspnea and angina in known AS. She had a history of arterial hypertension and her medication included beta-blockers, ARB, and a diuretic. TTE revealed a small left ventricle with marked hypertrophy (IVS 15 mm, PW 14 mm), hyperdynamic function (EF 66%) with no regional wall motion abnormalities, and SAM of AML with a dynamic LVOTO of 21 mmHg at rest and 42 mmHg after a Valsalva maneuver. There was also a mid-ventricular gradient of 20 mmHg. Severe AS was confirmed (maximum gradient 81 mmHg, mean gradient 51 mmHg). A coronary angiography showed patent coronary arteries.

**Figure 3:**
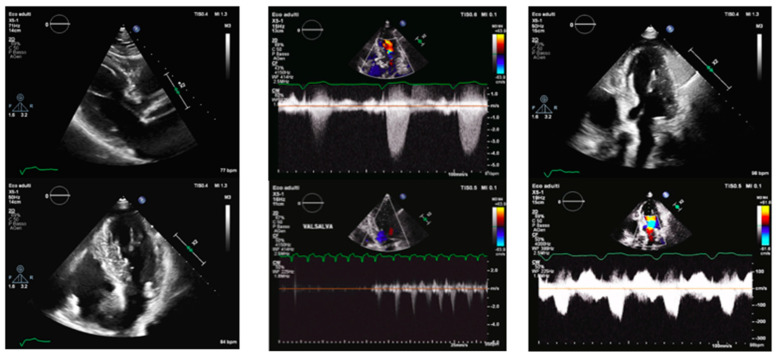
Transthoracic echocardiography of a 79-year-old woman before and after surgical aortic valve replacement and septal myectomy. Left panel. PLAX view (upper) and 4-chamber view (lower). IVS telediastolic diameter = 15 mm; LVEDVi 27 mL/m^2^. Central panel. Aortic valve peak velocity of 4.5 m/s (upper) with a mean gradient of 51 mmHg and dynamic LVOT gradient (lower) of 42 mmHg after the Valsalva maneuver. Right panel: after cardiac surgery. 4-chamber view (upper) and dynamic LVOT gradient of 11 mmHg (lower) after surgical aortic valve replacement. IVS: interventricular septum. LVEDVi: left ventricular end-diastolic volume indexed. LVOT: left ventricular outflow tract.

After the Heart Team discussion, in consideration of the patient’s age, good overall condition, and acceptable scores (STS 4.2%, and EUROscore II 6%), SAVR and a concurrent surgical myectomy according to Morrow’s procedure were preferred. Post-procedural evaluation showed a residual SAM of the chordal AML with a non-significant LVOT dynamic gradient (10 mmHg), a residual mid-ventricular gradient of 11 mmHg, mild MVR, and a well-functioning aortic bioprosthesis. The patient was discharged home with her current medication regimen, along with antiplatelet therapy and her follow-up has been excellent.

### 1.4. Case 4 (Figure 4)

An 80-year-old man with a known history of OHCM was admitted for severe exertional dyspnea. His genetic testing panel was negative for common genetic variants associated with hypertrophic cardiomyopathy. His medical treatment included beta-blockers and statin due to a history of hypertension and dyslipidemia. TTE revealed HLV (IVS of 18 mm, PW of 10 mm), with hyperdynamic function and an EF of 71%, with no regional wall motion abnormalities. Severe AS was noted with a peak gradient of 81 mmHg, a mean gradient of 50 mmHg, and a planimetric area of 0.6 cm^2^. SAM of the AML caused a dynamic LVOT gradient of 18 mmHg at rest and 40–45 mmHg after the Valsalva maneuver, with associated moderate MVR. A hemodynamic study showed no coronary artery stenosis and a mid-ventricular gradient of 35 mmHg. The case was discussed in the Heart Team and due to the high STS score (STS score of 6%, and EUROscore II was 7.2%), TAVI was proposed. The patient underwent TAVI with a 29 mm Evolut Pro+ bioprosthesis. Post-procedural TTE showed a residual LVOT gradient of 40 mmHg at rest and 65 mmHg after the Valsalva maneuver, with SAM of AML and mild MVR. Subsequently, due to the onset of a complete atrioventricular block, a dual-chamber pacemaker was implanted. Then, a therapy of increasing doses of oral metoprolol up to 50 mg twice a day resulted in a decrease of LVOTO and improvement of symptoms. The patient is currently doing well, and his follow-up has been excellent.

**Figure 4:**
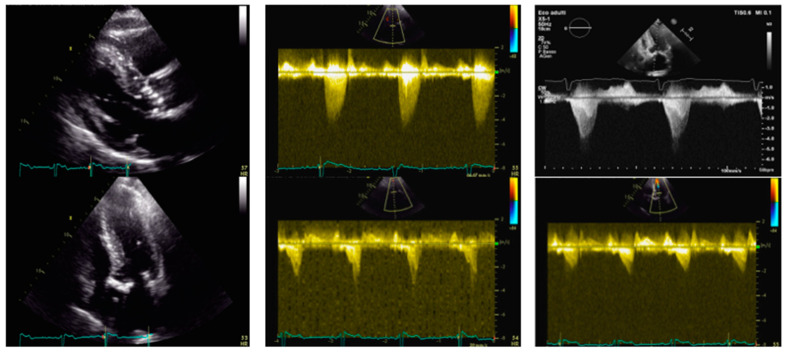
Transthoracic echocardiography of an 80-year-old man before and after aortic valve percutaneous replacement and pacemaker implantation. Left panel. PLAX view (upper) and 4-chamber view (lower). IVS telediastolic diameter = 18 mm; LVEDVi = 54 mL/m^2^. Central panel. Aortic valve peak velocity of 4.5 m/s (upper) with a mean gradient of 50 mmHg and dynamic LVOT gradient (lower) of 45 mmHg after the Valsalva maneuver. Right panel after TAVI. Increased dynamic LVOT gradient after TAVI (upper) of 65 mmHg after the Valsalva maneuver, which is reduced after pacemaker implantation (lower). IVS: interventricular septum. LVEDVi: left ventricular end-diastolic volume indexed. LVOT: left ventricular outflow tract. TAVI: transcatheter aortic valve implantation.

### 1.5. Suicide Left Ventricle: Pathophysiology and Clinical Implications

The term SLV expresses a severe form of left ventricular dysfunction, characterized by hemodynamic instability caused by dynamic obstruction of the LVOT after a sudden decrease in the cardiac afterload [7]. This situation can arise in patients who have undergone procedures to relieve AS such as TAVI or SAVR [8,9]. The SLV is characterized by an acute decrease in left ventricular afterload due to the sudden removal of the aortic valve obstruction, leading to a dramatic drop in left ventricular performance. The phenomenon is particularly concerning in patients with HLV, where the hypertrophied myocardium and associated diastolic dysfunction can exacerbate the situation [10,11].

**Figure 5:**
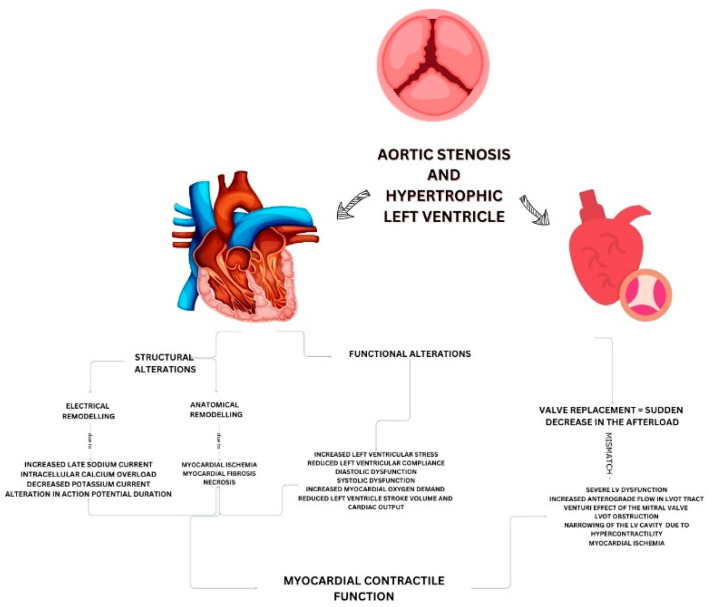
Pathophysiology of suicide left ventricle in patients with dynamic left ventricular outflow tract (LVOT) obstruction and aortic stenosis.

The pathophysiology of SLV involves a complex interplay of factors and a mismatch between the heart structure and the sudden change in pressure following valve replacement both percutaneously and surgically (Figure 5) [8,9,12]. In patients with severe HLV and AS, the heart adapts to the high afterload condition imposed by the AS [13,14]. This adaptation includes structural changes in the myocardium and alterations in ventricular compliance. When a competent bioprosthetic valve suddenly replaces the stenotic valve, the afterload is acutely reduced. This rapid change can overwhelm the left ventricle, leading to a mismatch between the myocardial contractile function and the new hemodynamic environment, resulting in severe left ventricular dysfunction [11]. After the LV is relieved of the pressure burden, due to increased blood flow, it starts to contract forcefully. Due to increased anterograde flow in the LVOT, the anterior mitral valve will be suctioned, as a Venturi effect, causing LVOTO [15]. Moreover, the hypercontractility is causing the narrowing of the LV cavity and subsequent LVOTO which impedes the anterograde blood flow. Increased inotropy and LVOTO can cause myocardial ischemia, further compromising the LV function and resulting in cardiogenic shock [8,9,10]. 

Furthermore, LVH associated with AS can sometimes be attributed to factors beyond the valvular disease itself. Concomitant conditions like sarcomeric HOCM and amyloidosis can also contribute to LVH, underscoring the necessity of a thorough evaluation in such patients [16,17]. Multimodality cardiac imaging plays a crucial role in this diagnostic process. A recent review by Vikash J et al. offers an in-depth review of diagnosing cardiac amyloidosis in the context of AS, highlighting the latest diagnostic techniques and proposing new algorithms to help clinicians accurately identify and manage cases where both conditions are present [16]. Cardiac MRI remains the gold standard for assessing myocardial pathology in patients with a high suspicion of HOCM. Recent studies emphasize the importance of using cardiac MRI to differentiate HOCM phenocopies, ensuring precise diagnosis and appropriate treatment [17].

The SLV clinical spectrum can manifest as acute heart failure, cardiogenic shock, and even death if not promptly recognized and managed [5,6,7,8,9,10,18,19]. The clinical presentation may include hypotension, low cardiac output, and signs of end-organ hypoperfusion. Diagnostic modalities such as echocardiography are crucial for identifying the reduced left ventricular function and guiding therapeutic interventions [20].

### 1.6. Management of Suicide Left Ventricle

Managing SLV requires a multifaceted and exhaustive approach. The primary goals are to stabilize the patient hemodynamically and to optimize left ventricular function. This could be achieved by the principle “from bench to bedside”, emphasizing the pathophysiological aspects focusing on the cellular level to its application to patient care. The first change after the exclusion of the aortic obstacle is the acute drop of the cardiac afterload, which led to cardiac hypercontractility. Therefore, the first logical step is to increase the cardiac afterload and this is achieved with vasopressor agents [21]. 

When selecting vasopressors to manage conditions like shock or severe hypotension, the primary goal is to increase afterload and support coronary perfusion while minimizing the impact on myocardial oxygen demand [21,22]. Phenylephrine is often considered the ideal choice due to its selective alpha-1 agonist activity, which increases systemic vascular resistance without affecting heart rate or myocardial contractility. This makes it effective in boosting afterload and maintaining coronary perfusion pressure. However, its intravenous formulation is not universally available, limiting its use in some regions [22].

Norepinephrine, which has both alpha-1 and beta-1 adrenergic effects, is a more commonly used alternative. It increases afterload through its alpha-1 activity while also enhancing cardiac contractility to a lesser extent via its beta-1 effects. This dual action makes norepinephrine a preferred option when there is a need to support both afterload and cardiac output, such as in cases of SLV [22].

In contrast, inotropes like dobutamine, which can increase heart rate and myocardial oxygen demand, are typically avoided in SLV. These agents may exacerbate acute heart failure by further compromising coronary perfusion, thus worsening the patient’s condition. Therefore, careful consideration is required when selecting the appropriate vasopressor or inotrope to balance the increasing afterload and maintaining myocardial health [22]. 

Even if it is counterintuitive, beta-blockers due to their negative inotropism, can reduce this effect [23]. 

In managing conditions like SLV, the preferred beta-blockers effectively lower heart rate and myocardial contractility without causing significant hypotension [24]. Esmolol is often the first choice due to its ultra-short-acting properties, which allow for precise titration and rapid discontinuation if necessary. This makes esmolol especially useful for managing dynamic outflow tract obstruction in acute settings, as it provides effective control without long-lasting effects [23,24].

Metoprolol is another commonly used cardioselective beta-blocker. Although it has a longer duration of action compared to esmolol, metoprolol is still relatively easy to titrate. It effectively reduces heart rate and myocardial contractility, thereby alleviating outflow tract obstruction while managing SLV. Both esmolol and metoprolol help in balancing the need for reduced cardiac workload with the requirement to maintain stable blood pressure levels [23,24]. However, they should be used with caution. Initial management includes aggressive volemic support, to prevent LV collapse, and when necessary, the use of specific drugs to maintain adequate blood pressure [7,8,9,10]. Right ventricle pacing could be used as a bailout therapy, by inducing ventricular asynchronism and subsequent decrease of intraventricular gradient [8]. In extreme cases, even in an acute setting, percutaneous correction of the mitral valve defect or septal alcoholization can be considered to improve patient outcomes. Mechanical circulatory support, such as extracorporeal membrane oxygenation (ECMO), may be necessary in severe cases, as depicted in several case report studies [8,9,10,18] 

However, there is scarce data regarding the best management for this population. The current literature includes several clinical studies and case reports that provide valuable insights into the occurrence and management of SLV [8,9,10,18,25]. The most relevant data from the literature are summarized in Table 1. For instance, a recent case report details the clinical journey of a patient with HLV and AS who developed SLV post-TAVI [23]. The report emphasizes the importance of recognizing early signs of left ventricular dysfunction and the prompt initiation of supportive therapies, highlighting the complexity of managing such patients and underscoring the need for individualized treatment plans. Similarly, another case report describes the management of a patient with severe HLV who underwent TAVI [26]. These case reports are instrumental in enhancing our understanding of SLV and guiding clinical practice. 

In our first reported case, intensive administration of vasopressor drugs was required due to severe refractory hypotension, and dobutamine was also initiated to support cardiac function. Additionally, a temporary pacing lead was implanted because of the development of a transient atrioventricular (AV) block.

In comparison to the case reported by Hellmuth S et al. [8], our intervention did not result in an improved hemodynamic profile or a significant reduction in intracardiac gradients as right ventricular pacing was temporary. This discrepancy highlights that while right ventricular pacing was necessary to address the transient complete AV block, it was not considered that it could improve the intracardiac gradients in our patient. Furthermore, our patient experienced acute hemodynamic deterioration following the deployment of the aortic prosthesis, whereas the previously mentioned case report noted late LVOTO after TAVI. Considering the severe mitral regurgitation, secondary to SLV, a percutaneous intervention for mitral valve correction was proposed but not performed due to the infectious condition and the concomitant unstable hemodynamic balance. 

**Figure 6:**
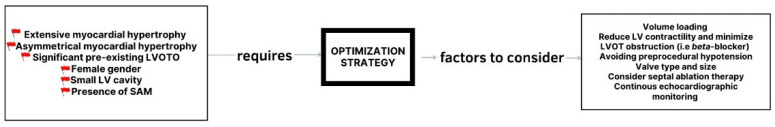
Red flags of suicide left ventricle and proposed strategy for hemodynamic optimization in patients with aortic stenosis and dynamic left ventricle outflow tract (LVOT) obstruction.

In the second case, firstly the TAVI procedure was performed preparing the patient with fluid-challenging and beta-blocking agents, secondly, the transcatheter septal reduction was performed, which acutely reduced the LVOTO gradient. As in a previous case report (Sorajja A et al.), catheter-based alcohol septal ablation was successful in reducing intracardiac gradients, but this procedure is highly dependent on the coronary anatomy, which was favorable in both cases [12]. Subsequently, we learned to recognize certain characteristics as red flags (Figure 6): a small left ventricle, significant septal hypertrophy, pre-existing SAM of the AML before the procedure, and female sex. 

Therefore, in the third case, we prevented the SLV by proposing surgical myectomy and subsequent AVR, especially considering the absence of comorbidities in the patient. The third reported clinical case is in line with the clinical characteristics of a recent observational study regarding the clinical outcomes in patients with both obstructive hypertrophic cardiomyopathy and severe aortic stenosis undergoing surgical myectomy and SAVR [6].

In the last case, pacemaker implantation, although it was done for advanced atrioventricular block, induced contraction desynchrony, which allowed the reduction of the intraventricular gradient. In cases of SLV managed with right apical pacing, the altered activation pattern of myocardial depolarization begins with ventricular depolarization at the right ventricular apex. This pacing approach induces a left bundle branch block, which causes paradoxical motion of the septum away from the anterior mitral valve leaflet. As a result, this mechanism reduces the dynamic outflow obstruction. This pattern of pacing and its effects on septal motion is consistent with findings from other SLV cases where right apical pacing has been employed [8].

### 1.7. Preventive Strategies and Best Practices

Preventive strategies are paramount in reducing the incidence and development of SLV. The female gender is considered a risk factor for SLV, primarily due to anatomical and physiological differences that can influence the heart’s response to abrupt changes in hemodynamics [9]. Women typically have smaller left ventricular cavities compared to men [28,29,30]. This can result in a more pronounced reduction in ventricular size and volume when afterload is suddenly decreased. Due to smaller ventricular volumes, women might be more sensitive to changes in intravascular volume and preload. This sensitivity can exacerbate the effects of rapid decompression of the left ventricle post-procedure. Moreover, there are recent findings that suggest gender-related differences in myocardial structure and function [28]. Women may have different patterns of myocardial hypertrophy and fibrosis, which can affect the heart’s adaptability to sudden changes in afterload [29]. Therefore, tailoring perioperative management strategies to mitigate the risk of SLV in female patients undergoing procedures for AS. 

Another critical aspect of prevention is the careful selection of patients for AVR. Echocardiography is the most important imaging techniques that ensure sufficient information for these patients, helping identify the red flags for this pathology [30]. Therefore, patients with extensive myocardial hypertrophy (more than 15 mm) or asymmetrical myocardial hypertrophy, as well as significant pre-existing LVOTO are at higher risk for SLV and may require alternative therapeutic approaches or enhanced peri-procedural monitoring [9]. As depicted in our case series, paradoxical low-flow low-gradient AS was associated with unfavorable outcomes, and this emphasizes the importance of a comprehensive echocardiographic evaluation [30]. Moreover, certain measures can be employed during the procedure, such as volume loading and aggressive bradycardia through beta-blocking agents to promote left ventricular filling.

Several case reports in the literature detail various strategies for managing patients with both AS and LVOTO. These strategies include combining SAVR with septal myectomy or, for high-surgical-risk patients, a two-step approach involving alcohol septal ablation followed by TAVR after 4–8 weeks [6,31,32]. Furthermore, TAVI combined with Percutaneous Intramyocardial Septal Radiofrequency Ablation (PIMSRA) which is an innovative, minimally invasive therapy for patients with both AS and obstructive HOCM, is expanding the treatment options for these situations [27].

Intra-procedural techniques, including the use of right ventricular pacing to temporarily reduce myocardial contractility and lower the intracardiac gradient, along with meticulous blood pressure management, can help mitigate the risk of SLV [16,20]. 

Post-procedural monitoring should focus on early detection of left ventricular dysfunction and prompt initiation of supportive measures. Multidisciplinary care involving cardiologists, interventional echocardiographers, cardiac surgeons, and critical care specialists is essential for optimizing patient outcomes.

### 1.8. Long-Term Outcomes and Prognosis of Patients with Suicide Left Ventricle

The long-term outcomes of patients who develop SLV post-TAVI are variable and depend on several factors, including the severity of left ventricular dysfunction, the effectiveness of initial management, and the presence of underlying comorbidities [6]. Some patients may recover left ventricular function with appropriate treatment, while others may experience persistent cardiac dysfunction and require long-term heart failure management.

Shenouda et al. highlight the need for personalized care plans and regular monitoring to address the evolving clinical needs of these patients. Ongoing follow-up and management are crucial to ensure optimal long-term outcomes [10].

The current literature provides valuable insights into the occurrence and management of SLV. Clinical studies and case reports emphasize the importance of recognizing early signs of left ventricular dysfunction and initiating prompt supportive therapies [8,9,10,16]. Preventive strategies, including careful patient selection, meticulous procedural planning, and vigilant post-procedural monitoring, are essential in reducing the incidence of SLV.

### 1.9. Gaps in Knowledge and Future Directions

Despite significant advances in managing patients undergoing AVR, several knowledge gaps remain regarding outcomes and their impact on post-AVR prognosis. The cellular and molecular mechanisms driving LV remodeling and dysfunction in this context are still not fully understood. There is no consensus on the optimal timing for AVR in patients with varying degrees of LVH, as delaying the procedure might lead to irreversible myocardial damage, however, early intervention could pose its own risks. More research is needed to elucidate the relationship between pre-operative LV conditions and postoperative outcomes.

Additionally, the factors predicting LV recovery after AVR are not well-established. While some patients experience complete recovery of LV function, others do not, and predictive models for identifying those who will benefit most from AVR need further refinement. The role of imaging biomarkers, such as myocardial strain and fibrosis quantification via MRI, requires additional investigation.

Identifying biomarkers that can predict early LV dysfunction post-AVR and distinguish between reversible and irreversible damage could enhance patient selection and timing for intervention. Large-scale, long-term studies are essential to track patients with varying degrees of LV dysfunction after AVR, providing a clearer understanding of the natural course of LV function and its correlation with patient outcomes.

## 2. Conclusions

The phenomenon of SLV post-TAVI presents a significant clinical challenge, particularly in patients with HF and concomitant AS. Understanding the pathophysiological mechanisms underlying SLV is crucial for developing effective management and prevention strategies. The current literature highlights the need for a comprehensive approach to these patients, incorporating detailed pre-procedural assessment, careful procedural execution, and vigilant post-procedural monitoring. Echocardiography is crucial in all stages: preoperatively, to identify potential red flags that may influence subsequent therapeutic decisions; intraprocedural, to monitor high-risk patients and detect SLV; and postoperatively, to confirm the resolution of the condition and assess the applicability of certain therapeutic options. As our understanding of SLV evolves, ongoing research and clinical vigilance will be essential in improving outcomes for these complex cardiac patients.

The integration of multidisciplinary care, the evaluation in the Heart Team, and personalized treatment plans is paramount in addressing the complex needs of these patients and improving their long-term outcomes. Clinicians must remain vigilant for signs of SLV, employ preventive strategies diligently, and be prepared to manage this serious complication should it arise.

## Figures and Tables

**Table 1:** Key literature insights on suicide left ventricle related to aortic valve replacement.

Authors	Title of Article	Type of Article	Cohort Study	Management
Sorajjia P, et al., 2012 [12]	Alcohol septal ablation after transcatheter aortic valve implantation: The dynamic nature of left outflow tract obstruction	Case report	One patient	Alcohol septal ablation
M.Y. Desay et al., 2021 [6]	Outcomes in Patients with Obstructive Hypertrophic Cardiomyopathy and Concomitant Aortic Stenosis Undergoing Surgical Myectomy and Aortic Valve Replacement	Observational	191 patients with hypertrophic cardiomyopathy and moderate/severe aortic stenosis	Myectomy and surgical aortic valve replacement
H.S Weich et al., 2021 [8]	Dynamic Left Ventricular Outflow Tract Obstruction Post-Transcatheter Aortic Valve Replacement	Case report	One patient	Apical right ventricle pacing
Y.Li et al., 2021 [27]	Case report: Minimally Invasive Therapy by Transcatheter Aortic Valve Replacement and Percutaneous Intramyocardial Septal Radiofrequency Ablation for a Patient with Aortic Stenosis Combined with Hypertrophic Obstructive Cardiomyopathy: Two year follow-Up Results	Case report	One patient	Percutaneous intramyocardial septal radiofrequency ablation
P.A Lioufas et al., 2022 [18]	Unexpected suicide left ventricle post-surgical aortic valve replacement requiring veno-arterial extracorporeal membrane oxygenation support despite gold-standard therapy: a case report	Case report	One patient	Alcohol septal ablation followed by veno-arterial extracorporeal membrane oxygenation
I.B. Dor et al., 2022 [26]	A Word of Caution Before Treating Aortic Stenosis in Patients With Concomitant LVOT Obstruction	Case report	Three patients	Alcohol septal ablation and beta-blocker
H. Chraibi et al., 2023 [7]	Conservative Management of Suicide Left Ventricle After Surgical Aortic Valve Replacement	Case report	One patient	Conservatively—Volume loading and oral beta-blocker
L. Koliastasis et al., 2023 [23]	Overcoming the Obstacle of Suicide Left Ventricle After Transcatheter Aortic Valve Replacement Phenomenon	Case report	One patient	Vasopressors and Dual Chamber pacemaker implantation
D. Barzallo et al., 2024 [9]	Acute Hemodynamic Compromise After Transcatheter Aortic Valve Replacement Due to Dynamic Left Ventricle Obstruction: A systematic review	Systematic review	25 publications	Advanced therapies required in nearly 65% of patients

## Data Availability

No new data were created or analyzed in this study. Data sharing is not applicable to this article.
